# Perforated jejunal diverticula in a young woman: A case report

**DOI:** 10.1016/j.ijscr.2021.105838

**Published:** 2021-03-26

**Authors:** Sem F. Hardon, Frank C. den Boer, Tarik Aallali, Gerwin A. Fransen, Sandra Muller

**Affiliations:** a Department of Surgery, Zaans Medical Center, Zaandam, the Netherlands; b Department of Surgery, Amsterdam UMC – VU University Medical Center, Amsterdam, the Netherlands; c Symbiant Pathology Expert Centre, Hoorn/Zaandam, the Netherlands; d Department of Radiology, Zaans Medical Center, Zaandam, the Netherlands

**Keywords:** JD, jejunal diverticulosis, Acute abdomen, Jejunal diverticulosis, Perforation, Case report, Surgery

## Abstract

**Introduction and importance:**

Small bowel diverticulosis is a rare condition. It usually remains asymptomatic and undiscovered, until potentially severe complications such as diverticulitis or even perforation occur. We present an unusual case and discuss the pathophysiology, diagnostics strategies, and possible surgical intervention.

**Case presentation:**

A young woman was referred to our emergency department suffering from acute abdominal pain. A computed tomography scan showed signs of small bowel perforation of unknown origin. Exploratory laparotomy revealed multiple perforated jejunal diverticula (JD). The patient underwent segmental resection of the affected jejunum followed by primary anastomosis. Pathological examination confirmed the diagnosis of perforated JD.

**Conclusions:**

Due to its rarity and variable clinical presentation, it can sometimes be challenging to diagnose this potentially life-threatening condition. If the bowel is suspected from perforation; segmental resection is the treatment of choice, preferably followed by direct restoration of the bowel continuity.

## Highlights

• Small bowel diverticula: a rare condition with severe complications.
• We present a rare case of a young, female patient.
• SBD should be considered a differential diagnosis of acute abdominal pain.
• Early diagnosis and treatment is mandatory to reduce the risk for complications.
• Recommended treatment is segmental resection followed by primary anastomosis.

## 1 Background

Small bowel diverticulosis, in contrast to colonic diverticular disease, is a rare condition [1,2]. In particular, acquired diverticula found in the jejunum or ileum (referred to as jejunoileal diverticular disease) are uncommon [3]. Likewise other diverticular diseases, its incidence increases with age. The reported incidence varies between 0.06–2.3% [1,2,4,5]. Most cases remain asymptomatic and therefore undiscovered unless complications such as diverticulitis or even perforation occur [6]. If so, symptoms are mostly nonspecific and it can be challenging to differentiate them from more common causes of acute abdominal pain. Hence, these lesions may rise a significant dilemma concerning adequate diagnosis and treatment. We present an unusual case of a young female patient suffering from complicated JD, who needed acute surgical treatment. After a brief case presentation, strategies for clinical diagnosis and optimal surgical treatment will be discussed.

## 2 Case presentation

A 37-year-old woman with a history of laparotomic intestinal derotation during childhood was referred to our emergency department because of sudden onset pain in the lower and upper left abdomen. There was no relevant drug or family history. The pain was worsening over the past 48 h and now radiating to the right lower quadrant. The patient reported nausea without vomiting and the absence of stool since the start of the pain. The vital signs were slightly abnormal, with a sinus rhythm of 95 bpm, blood pressure of 121/90 mmHg, and a body temperature of 37.4 C. On physical examination, sparse peristalsis was auscultated over the abdomen. The pain was most prominently in the left upper quadrant, with guarding and rebound tenderness.

**Fig. 1:**
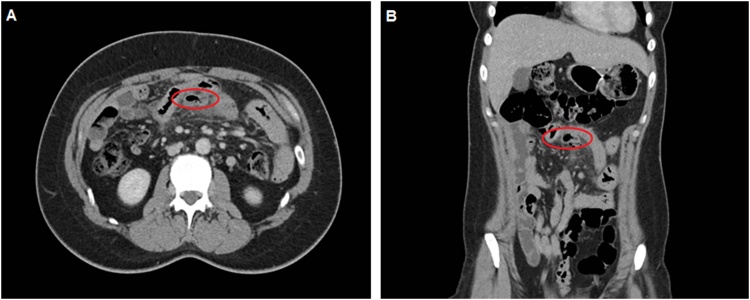
Axial (Fig. 1A) and coronal (Fig. 1B) post-contrast abdominal computed tomography scan, portal venous phase. Caudal and dorsal of the small bowel loop is a diverticulum, connected with the small bowel loop, with adjacent fatty infiltration and gas bubbles in the mesentery, representing small bowel diverticulitis with possible perforation.

Laboratory results showed a leukocyte count of 19 × 10E9/l and a C-reactive protein level of 317 mg/l. A computed tomography scan (Fig. 1) showed signs of air centrally in the mesentery, just cranial from the umbilicus, surrounded by enlarged lymph nodes. The radiology report described a diverticulum in the small bowel, most likely a Meckel’s. The suspicion of a perforated hollow viscus was increased by the presence of free intraperitoneal fluid just behind the anterior abdominal wall. No further abnormalities were detected, especially the absence of colonic diverticula or other (non)inflammatory processes.

The patient underwent a laparotomy (performed by SM and SH) which macroscopically revealed the diagnosis of perforated JD. Multiple JD were revealed, of which at least one was perforated. A segmental (80 cm) resection of the affected jejunum was carried out, followed by a side-to-side hand-sewn primary anastomosis. Postoperatively, a 5-day course of antibiotics was administered. The patient was able to tolerate oral intake after the removal of the nasogastric tube. On day six, the patient was discharged home. At the two-week follow-up visit, she was fully recovered. No adverse or unanticipated events were registered 6 months after.

**Fig. 2:**
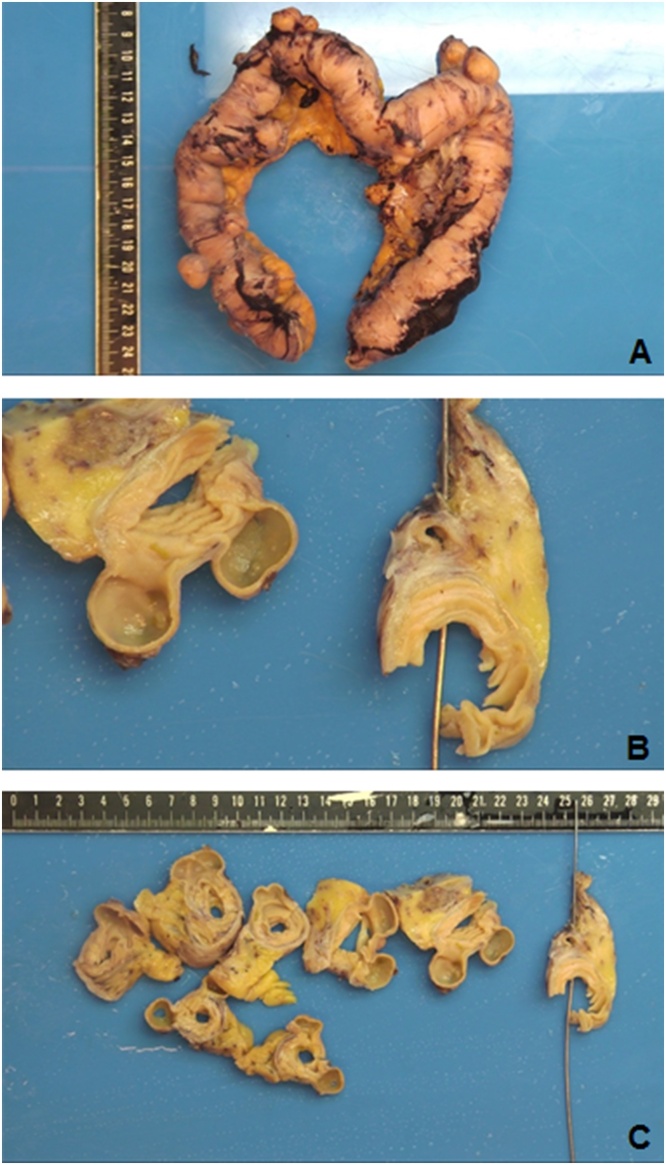
Gross specimen of jejunum with numerous diverticula and reactive changes of the bowel wall; thickened muscle layer and hemorrhagic serosa (Fig. 2A). Cross section shows outpouching of the mucosa and submucosa through the bowel wall with a perforation (Fig. 2B & C).

**Fig. 3:**
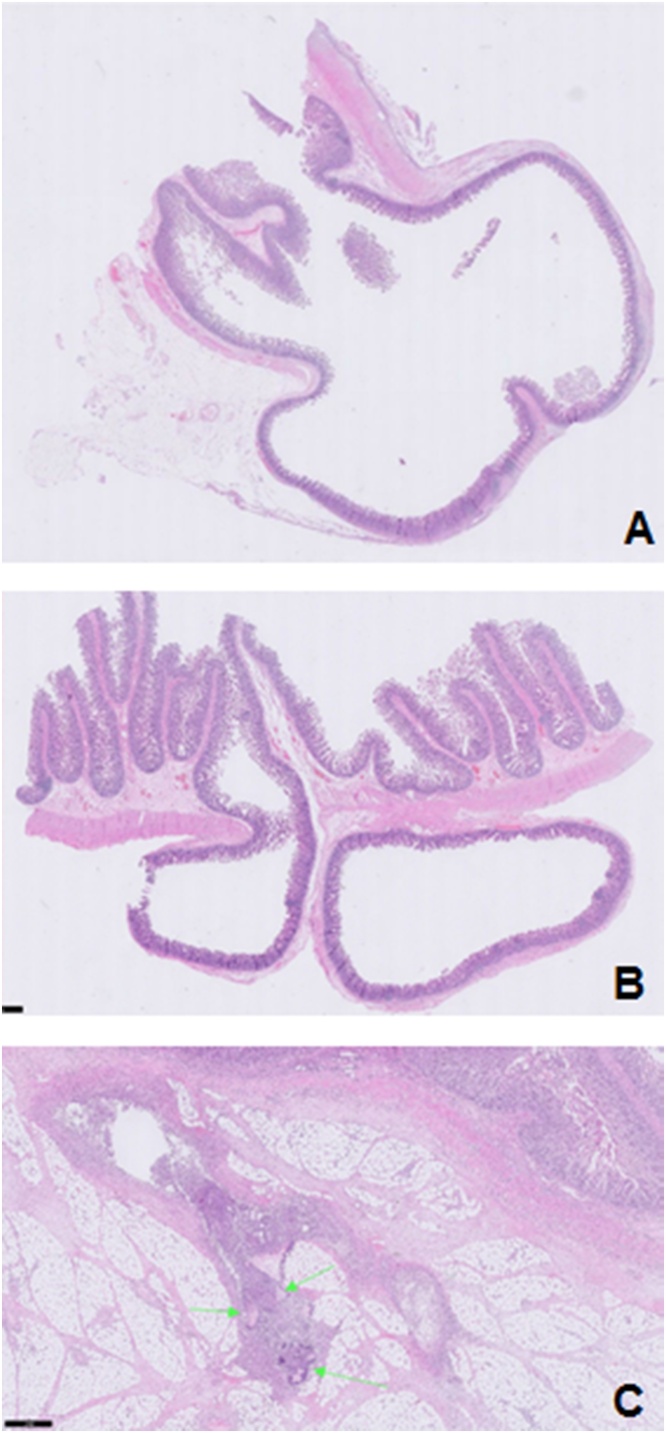
The histologic section shows flask-shaped mucosal outpouchings that extend deep into the bowel wall through the muscularis propria into the subserosal adipose tissue (Fig. 3A & B). Fig. 3 shows perforated diverticulitis complicated by abscess formation with suppurative inflammation to faecal material (indicated by the green arrows).

Histopathological examination (Fig. 2, Fig. 3) confirmed the diagnosis and showed multiple, small, and medium-sized, flask-like invaginations present through the muscularis propria, filled with mucin or stool but easily emptied, bulged into the serosa. Central perforation of the diverticular part with accompanying inflammation and fibrin plaques along the serosa. There is a suppurative inflammation of the diverticula and peridiverticular abscess formation with foreign-body giant cell reaction to faecal material (green arrows in Fig. 3C) with fat necrosis. This work has been reported in line with the SCARE 2020 criteria [28].

## 3 Discussion

Small bowel perforations caused by JID are rare [7,8]. Considering the severity of this condition and the likelihood of being mistaken for other more common causes of acute abdominal pain, every physician should be aware of the pathophysiological aspects, preferred diagnostics, and the most appropriate treatment. As Coulier et al. reported previously, the vast majority manifests itself in the elderly, and JD is seldom found in patients under the age of 40(1, 5). Males, when compared to females, are affected twice as frequently [1,4,9,10]. In contrast to the vast majority of cases described in the literature [6], which predominantly reports elderly, male patients, we present a case of a young woman with JD. The presentation, diagnosis, and preferred treatment of JD have only been described to a limited extent in the literature.

### 3.1 Pathophysiology

Despite true diverticula like the Meckel’s diverticulum, all diverticula are considered to be acquired pseudodiverticula; mucosal and submucosal protrusion through the muscular tissue of the gastrointestinal tract [1,6,11]. Their exact etiology remains unknown. However, these protrusions are thought to be the result of intestinal dyskinesia due to motor dysfunction of muscularis propria (e.g. Auerbach's plexus) [[12], [13], [14]]. Another hypothesis focuses on dysmotility caused by a lack of dietary fibers [15]. As a result, intraluminal pressure increases, which is associated with the protrusion of false diverticula. These herniations tend to occur at the weakest point of the mesenteric border, where the blood vessels penetrate through the intestinal wall [7,16,17].

### 3.2 Treatment

Similar to the treatment of CDD, conservative medical treatment of SBD may be considered just to control diarrhea or mild anaemia [11]. If uncomplicated inflammation occurs, we suggest a regime of intravenous broad-spectrum antibiotics that covers enteric bacteria and close observation for the development of complications [[18], [19], [20]]. However, if medical management fails and the inflammation progresses or if signs of beginning abdominal sepsis are present (with or without the presence of peritonitis), surgical resection should be performed. It has been reported that some cases of localized, confined perforation or abscess without generalized peritonitis may be treated with either intravenous antibiotic therapy, CT-assisted percutaneous drainage, or laparoscopic lavage and drainage [21,22]. However, in the case of complicated jejunal diverticula, standard treatment should consist of exploratory laparotomy with segment resection of the affected bowel, followed by primary anastomosis or construction of an end enterostomy [[23], [24], [25]]. Other indications for surgical intervention include acute obstruction of the affected bowel and severe anaemia due to hemorrhage. If asymptomatic diverticula are discovered as an incidental imaging finding [26] or during surgery for other indications [12,27], there is, besides prevention of recurrent diverticulitis, no indication for resection. Diverticulectomy should only be performed in case of uncomplicated Meckel’s diverticula and only after exclusion of Crohn’s disease [11,23].

## 4 Conclusion

This case report demonstrates that not only elderly, male, patients can be affected by (complicated) JD. Failing to recognize and diagnose this disease timely might lead to severe complications, such as perforation. Therefore, this condition should be considered in the differential diagnosis of acute abdominal pain. If small bowel perforation is suspected, surgical resection followed by primary anastomosis is the treatment of choice.

## Declaration of Competing Interest

None.

## Sources of funding

None.

## Ethical approval

N/A.

## Consent

Written informed consent was obtained from the patient for publication of this case report and accompanying images. A copy of the written consent is available for review by the Editor-in-Chief of this journal on request.

## Author contribution

SH and SM admitted the patient and performed surgery. SH, TA, and GF collected patient data. SH, FB and SM desgined this report, reviewed the literature, analyzed data and drafted the manuscript. FB and SM made final approval of the manuscript.

## Registration of research studies

Not applicable.

## Guarantor

Sem F. Hardon, MD.

## Provenance and peer review

Not commissioned, externally peer-reviewed.
